# Surface temperature controls the pattern of post-earthquake landslide activity

**DOI:** 10.1038/s41598-022-04992-8

**Published:** 2022-01-19

**Authors:** Marco Loche, Gianvito Scaringi, Ali P. Yunus, Filippo Catani, Hakan Tanyaş, William Frodella, Xuanmei Fan, Luigi Lombardo

**Affiliations:** 1 Institute of Hydrogeology, Engineering Geology and Applied Geophysics, Faculty of Science, Charles University, Prague, Czech Republic; 2 State Key Laboratory of Geohazard Prevention and Geoenvironment Protection, Chengdu University of Technology, Chengdu, China; 3 Department of Geosciences, University of Padova, Padua, Italy; 4 Faculty of Geo-Information Science and Earth Observation (ITC), University of Twente, Enschede, The Netherlands; 5 Department of Earth Sciences, University of Florence, Florence, Italy

**Keywords:** Natural hazards, Geomorphology

## Abstract

The patterns and controls of the transient enhanced landsliding that follows strong earthquakes remain elusive. Geostatistical models can provide clues on the underlying processes by identifying relationships with a number of physical variables. These models do not typically consider thermal information, even though temperature is known to affect the hydro-mechanical behavior of geomaterials, which, in turn, controls slope stability. Here, we develop a slope unit-based multitemporal susceptibility model for the epicentral region of the 2008 Wenchuan earthquake to explore how land surface temperature (LST) relates to landslide patterns over time. We find that LST can explain post-earthquake landsliding while it has no visible effect on the coseismic scene, which is dominated by the strong shaking. Specifically, as the landscape progressively recovers and landslide rates decay to pre-earthquake levels, a positive relationship between LST and landslide persistence emerges. This seems consistent with the action of healing processes, capable of restoring the thermal sensitivity of the slope material after the seismic disturbance. Although analyses in other contexts (not necessarily seismic) are warranted, we advocate for the inclusion of thermal information in geostatistical modeling as it can help form a more physically consistent picture of slope stability controls.

## Introduction

Strong earthquakes in steep mountains generate abundant mass wasting through earthquake-induced landslides^1^ (EQILs), ultimately driving landscape evolution^2–4^. Landslide rates peak upon the mainshock but decay to background levels within years, even for the strongest shaking^5,6^, leaving deposits of unconsolidated debris and weakened slopes^7–9^. The factors controlling EQIL patterns and the physical processes responsible for their fading activity are not fully understood, partly owing to the dynamic nature of the postseismic landscape^7^ and the scarcity of multi-temporal inventories.

Landslide susceptibility modeling (LSM) can help identify these processes by explaining landslide spatial patterns via statistical or physically-based relationships with morphological, lithological, seismo-tectonic, hydro-climatic, land use, and vegetation-related variables^10,11^. Information on precipitation is typically accounted for because precipitation is a recognized trigger and predisposing factor of landslides. Climatic trends also are considered via changes in precipitation and evapotranspiration^12–15^, while temperature oscillations and trends are not accounted for explicitly (as model covariates)^16,17^. That is, current LSM approaches do not consider temperature as a possible direct driver of slope instability, even under climate change scenarios, except when discriminating between liquid and solid precipitation^18^ or evaluating the depth of the active layer in cold climates.

This picture contrasts with experimental results and field evidence of a direct role of temperature in the hydro-mechanical behavior of geomaterials, also in typical ranges for the ground surface and near subsurface. Pioneering studies have linked the interplay of the thermal expansion of water and soil minerals, and changes in interparticle forces, to volume and pore water pressure changes in saturated soils^19^. Drained heating–cooling cycles in normally consolidated soils were seen to produce net shrinkage and stiffening, and hence an apparent preconsolidation^20^. A similar behavior was determined in unsaturated soils^21^. Soil compressibility is both stress- and temperature-dependent^19^. Higher temperatures typically imply larger volumetric and shear strains but lower plasticity and water retention capacity^22^, partly owing to the smaller viscosity of the pore water^23^. In undrained condition, a 20 °C rise in temperature can produce up to a 30% decrease in shear resistance in active clays^20^. In drained condition, high temperatures can enhance softening, as an elevated peak strength (resulting from the apparent preconsolidation) is followed by a sharp post-peak decay to a lowered ultimate strength^19,24^. Recent works, however, suggested a more complex picture, pointing out that the shear resistance can either increase or decrease with temperature depending on mineralogy and stress-thermal history^25^. A relationship was also observed among temperature, mineralogy (particularly the smectite content), and shear rate effects^26^, which may be able to control the potential for runaway motion in landslides.

At the slope scale, fracturing and disaggregation caused by combined thermal–hydraulic forcing are well-recognized drivers of weathering in rocks. Similar processes occur in stiff and swelling clays^27^. Discontinuities and shear zones can transfer pore water pressure changes to large depths^14,28,29^, as well as heat through water or air flow^30–32^. Destructuration, softening, and enhanced weathering in a deforming body may result in distinct thermal properties and hence surface temperatures compared with adjacent areas^33^. The difference may be further enhanced by a lack of vegetation. Temperature can also be related to soil moisture^34^ and evapotranspiration^35^, capable of changing landslide-triggering precipitation thresholds^14,36^. Examples of thermally controlled landslide slip zones have been reported, such as the Ru delle Roe^37^ and Touge^38^ landslides. In the latter case, landslide activity was explained as a function of temperature in the shear zone, consistently with experiments showing a 13% decrease in shear resistance upon cooling from 25 °C to 9 °C. However, thermo-hydro-mechanical models are rarely applied in slope stability assessments^35^, especially in non-freezing climates, even though modeling tools^39^, developed for specific engineering applications, could in principle be used.

Upscaling physically-based models to entire catchments is difficult as simplifications in boundary conditions, parameters, and models are often necessary^40–42^. Fully-coupled thermo-hydro-mechanical models have not been implemented at this scale, although partial or sequential couplings have been attempted^18,43^. Currently, geostatistical modeling is the only viable tool for evaluating the non-straightforward (yet possibly direct) effect of temperature on landslide patterns at the catchment scale.

The simple binary logistic regression, a generalized linear model, is widely used in LSM^10,11,44,45^. It assumes that landslide patterns behave according to a Bernoulli exponential distribution and that the influence of the covariates can be captured via linear relationships^46,47^. However, the linearity assumption may not hold or may not be sufficient to explain complex effects, such as those favoring the genesis of a large population of landslides, especially in postseismic periods. Therefore, a generalized additive model (GAM) could be a more suitable choice, as it allows for complex nonlinear relationships, and of which numerous examples already exist in the geomorphological literature^48–51^.

The way the geographic space is partitioned can unveil further insight. Most studies use regular lattices or pixel-based subdivisions^10,52,53^, whereas fewer rely on slope units (SUs)^54–56^. The former can be expressed at very fine resolution, yet they do not carry information on the landslide body or shape unless further elaboration^57^ is performed. On the other hand, SUs are physical entities that result from geomorphological processes shaping the landscape as much as landslides. The shape of a SU can be a relevant parameter in relation to landslide occurrences, yet it has seldomly been studied^54^.

**Figure 1:**
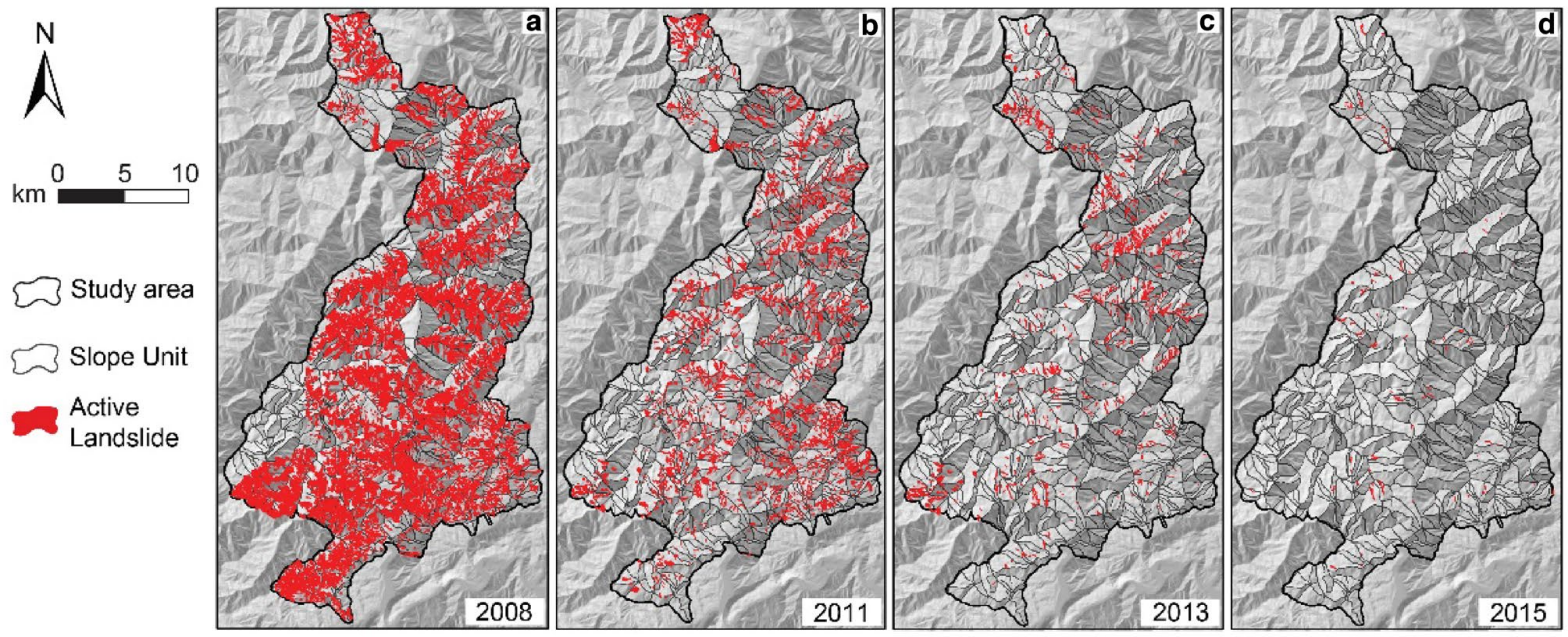
Active landslides in the WEER-MLI. (**a**) Coseismic scene and (**b–d**) three postseismic scenes showing the active landslides as polygons (data from Fan et al.^58^). A slope unit subdivision is also displayed [software: ArcGIS 10.5, www.arcgis.com].

All this considered, we chose to develop a LSM workflow using a GAM with SU partitioning and exploited the capabilities and data availability of Google Earth Engine (GEE). We accounted for thermal effects through land surface temperature (LST) data elaborated from the *Thermal InfraRed Sensor* (TIRS) of Landsat 7 and explored how these effects change over time in a rapidly evolving landslide-rich context. To this aim, we utilized the Wenchuan earthquake epicentral region multi-temporal landslide inventory (WEER-MLI)^58^ (Fig. 1) and accounted for geometric information at the SU level (slope, length, elongation) obtained from a detailed digital elevation model (DEM), together with a peak ground acceleration (PGA) map of the mainshock (see Methods).

## Results

### Fitting and trends

**Figure 2:**
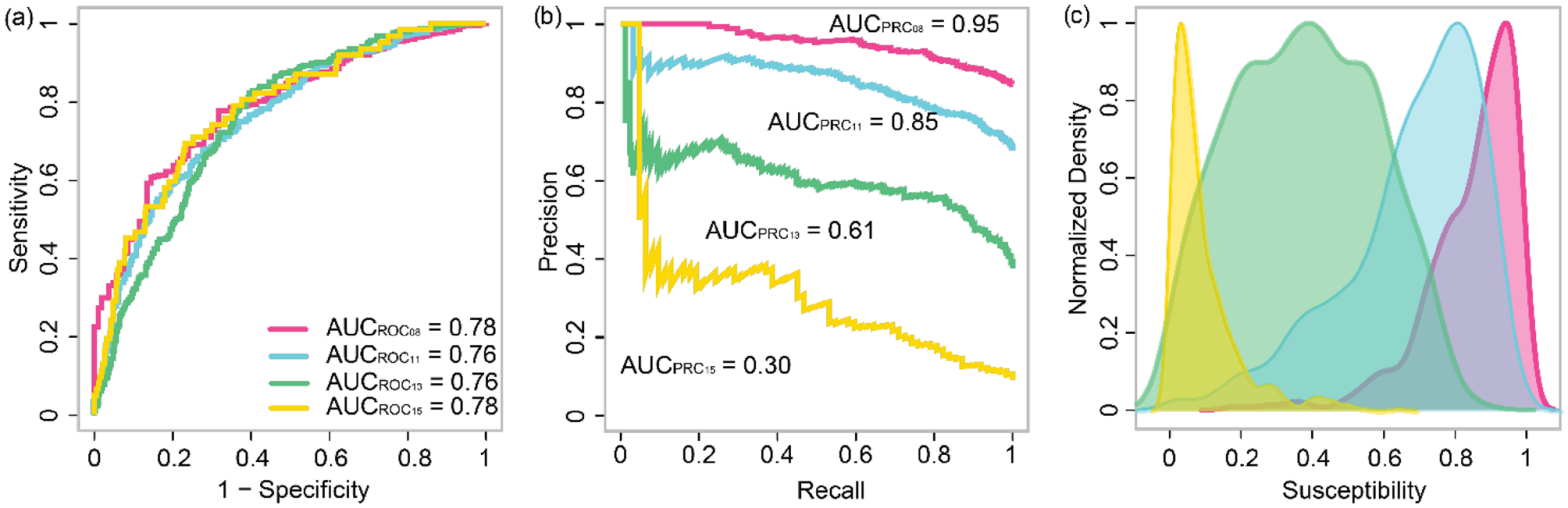
Model performance and susceptibility distribution. (**a**) ROC curves, (**b**) PR curves, and (**c**) probability density functions of landslide susceptibility for the coseismic (2008) and three postseismic (2011, 2013, 2015) scenes of the WEER-MLI [software: R 3.6.3, https://cloud.r-project.org/].

The fitting procedure returned a satisfactory result^59^ in terms of *receiver operating characteristic* (ROC) curves and their integral, the *area under the curve* (AUC), with values of 0.75–0.78 (Fig. 2a). In terms of *precision-recall* (PR) curves, the AUC decreases with time as the imbalance in the dataset increases, that is, as the amount of SUs with active landslides decreases (Fig. 2b).

Clearly, the model performance is influenced by the number of covariates, which we intentionally kept low to have the simplest possible model^44^ and facilitate a physical interpretation. Following this idea, we minimized the chance of interactions among the covariates (see the multicollinearity test in Figure S1, supplementary information) and chose, for instance, not to use those expressing the SU orientation. We did not consider the bedrock lithology as previous studies found that it had little influence on the coseismic^60^ and postseismic^7^ pictures in the epicentral region owing to its uniformity (mostly granite). Similarly, available precipitation metrics were not found to exert a prime control on the postseismic evolution of landslide activity^7^, owing to the smooth spatial changes of cumulative values observed in the study area. The triggering effect of precipitation can hardly be captured via satellite observations (cumulated over long periods) having a resolution (~ 10 km) that is worse than the spatial variability in storm precipitation, which is especially accentuated in mountainous contexts^58,61^. A similar observation could be made with respect to moisture data (controlling soil saturation and hence the mechanical strength): the resolution of available remote sensing products^62^ is insufficient to detect fine changes across SUs.

Consistently with the spike and decay of landslide activity—observed both qualitatively (Fig. 1) and quantitatively^1,5,6^—the probability density function (PDF) of landslide susceptibility attains the highest values in the coseismic scene, where most SUs show values closer to 1 (Fig. 2c). High landslide activity persists in the first postseismic scene (year 2011), with most SUs having susceptibility values larger than 0.6. The shape of the PDF does not change much from the coseismic scene, and this is consistent with the observation^5^ that changes in landslide activity between 2008 and 2011 appear stochastic^4^ and unrelated to specific landslide sizes or locations. The 2013 and 2015 maps yield significantly different PDF curves: approximately symmetric in 2013 (centered at 0.3) and pressed against 0 in 2015, with fewer SUs exhibiting larger susceptibility values. A similar right-tailed distribution was obtained by analyzing the inventory in terms of landslide size-frequency distributions^5,58^, as the largest (and infrequent) landslides remained active for a longer time^5^. In these landslides, the effect of healing processes takes a longer time to emerge^8^. Moreover, larger bodies may be associated with a lower mobilized friction^63^, conducive to longer/prolonged runouts.

### Effects of variables

**Figure 3:**
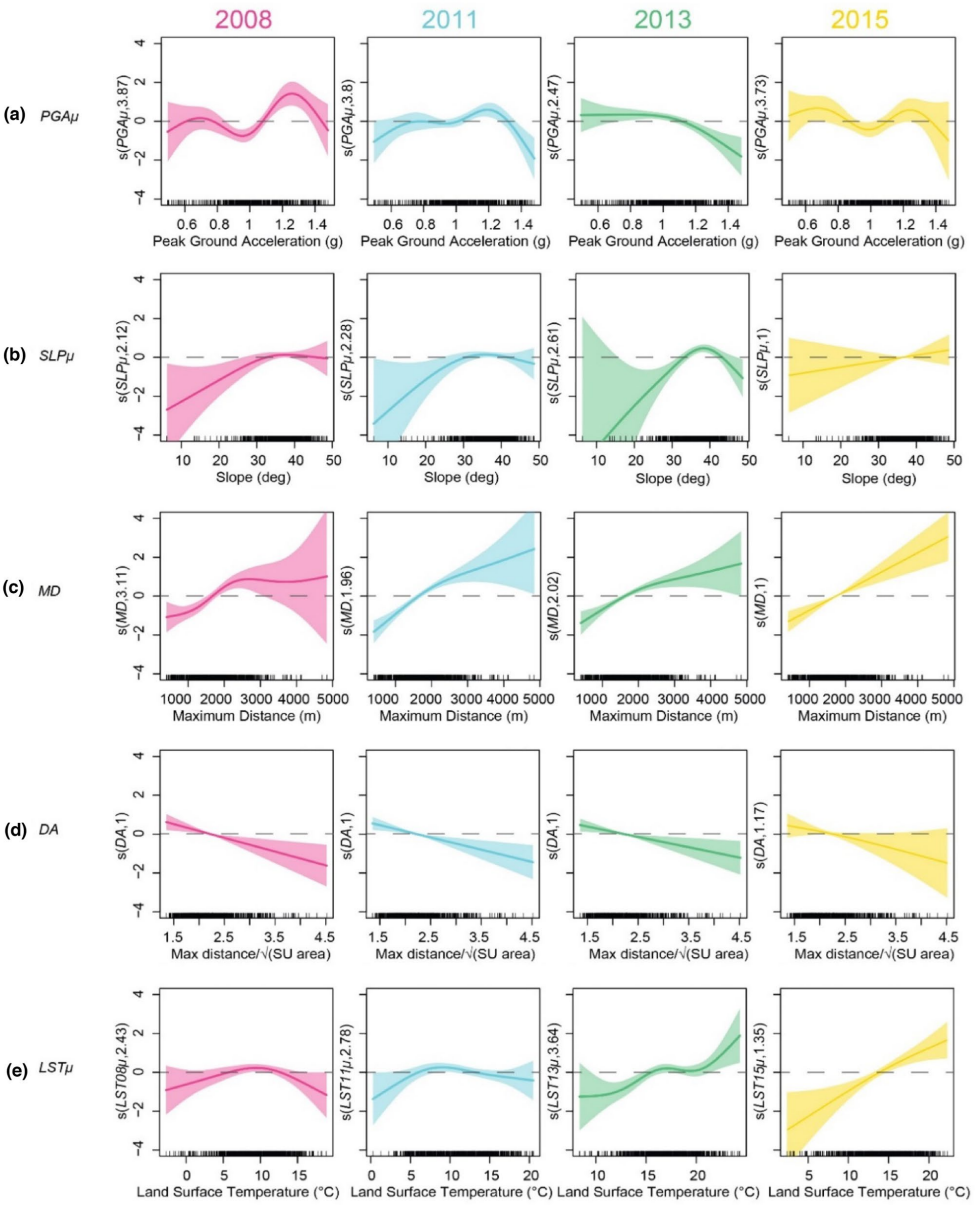
Variable effects of the model covariates over time. Effect of (**a**) mean peak ground acceleration, (**b**) mean slope, (**c**) maximum distance and (**d**) elongation of the SU, and (**e**) mean land surface temperature displayed as component smooth functions. The shaded areas are 95% confidence bands. The small vertical bars on the horizontal axes reflect the distribution of values across the SUs. The values in parentheses on the ordinates are the effective degrees of freedom, a proxy of the degree of non-linearity of the functions [software: R 3.6.3, https://cloud.r-project.org/].

Despite its intentional simplicity, our model offers some intriguing insight if one looks at the effects and uncertainties associated with each variable in each mapping year, and accounts for the density of information in the data ranges. The coseismic map is the richest in landslides. Most SUs are characterized by *PGAµ* values (Fig. 3a) in a narrow range (0.9–1.2 g). Within this range, a linear correlation can be seen, with larger values associated with higher susceptibility. Interestingly, the shape of the relationship persists in 2011 but is considerably attenuated. Moving to the 2013 and 2015 maps, the statistical significance in this range fades away. Indeed, the legacy of the strong ground shaking in terms of enhanced landsliding was reported to last less than a decade^7^. From 2008 to 2013, one can also see the emergence and widening of a range in which a significant negative correlation is evaluated (PGA*µ* >  ~ 1.1 g in 2013). This could be interpreted as a *flushing* effect of the strongest shaking, capable of clearing the slopes of landslide-susceptible material and exposing an underlying less weathered layer or even fresh bedrock.

The slope (here expressed as the average slope angle over a SU; *SLPµ*; Fig. 3b) affects the landslide occurrence/activity by controlling the balance between the destabilizing effect of gravity and the shear resistance of the material. Thus, gentle slopes are generally stable while steeper ones are increasingly susceptible to landsliding. However, very steep slopes are unlikely to have thick soil covers (bedrock outcrops are more likely) and water drains from them comparatively easily. Consequently, at a given time, the largest concentration of landslides should be expected in a defined range of slope angles, which in our case is centered around 35°, a value that should resemble the friction angle of the slope material^64,65^. It can be noticed, however, that the correlation is largely insignificant, suggesting that different slope angles in this range are not associated with significantly different susceptibility values, at least in the 2008 and 2011 maps. This is reasonable and can be attributed to a *saturation* effect of the very strong ground shaking, capable of triggering landslides on slopes with a wide range of inclinations, provided that sufficient mobilizable material is present. Interestingly, it can also be seen that the shape of the relationship persists through time (except in 2015, when the amount of SUs with active landslides was limited): strong ground shaking does not cancel gravity but renders its effect temporarily (statistically) insignificant.

Similar observations can be made for the two geomorphic metrics related to the SUs (Fig. 3c, d): their patterns remain consistent through time and do not show a dependence on external forcing (ground shaking or temperature variations). In particular, the length parameter *MD* plays consistently in favor of instability: longer—and, for a given shape, larger—SUs are more likely to contain active landslides. Conversely, the shape parameter *DA* exhibits a negative relationship in all years: large values of the parameter indicate elongated and, for a given length or area, narrow SUs. These observations are consistent with other SU-based susceptibility studies^54,66^. In narrow SUs, landsliding may be less likely owing to geomorphic constraints. A recent study^67^ on the shape of EQILs and rainfall-triggered landslides showed indeed that EQILs tend to be less elongated as their size increases, suggesting that narrow SUs may indeed hinder their development. However, the same study pointed out an opposite relationship for rainfall-triggered landslides, reflecting the long runout of large debris flows and the length of drainage paths over which flow accumulation and entrainment occur. This pattern is not reflected in our results as we explicitly excluded debris flows from the model.

Finally, the effect of LST emerges through time progressively (LST*µ*; Fig. 3e). In the 2008 and 2011 scenes, a statistically significant effect of temperature on landsliding cannot be evaluated. Again, this could be explained with a *saturation* effect of the PGA. However, as the landscape stabilizes and the legacy of the shaking fades, a significant positive correlation arises: slopes characterized by warmer surfaces are comparatively more susceptible to landsliding than the cooler ones. Interestingly, different from the morphometric parameters, both LST*µ* and PGA*µ* exhibit correlations that evolve in shape through time, suggesting that physical processes of slope healing are taking place at a speed that is conditioned by the value of these variables. In short, the model hints at longer healing times (and thus prolonged landslide activity) for slopes that did not suffer the strongest shaking (i.e., for which flushing was less likely to occur) and whose surface remained comparatively warmer throughout the years.

**Figure 4:**
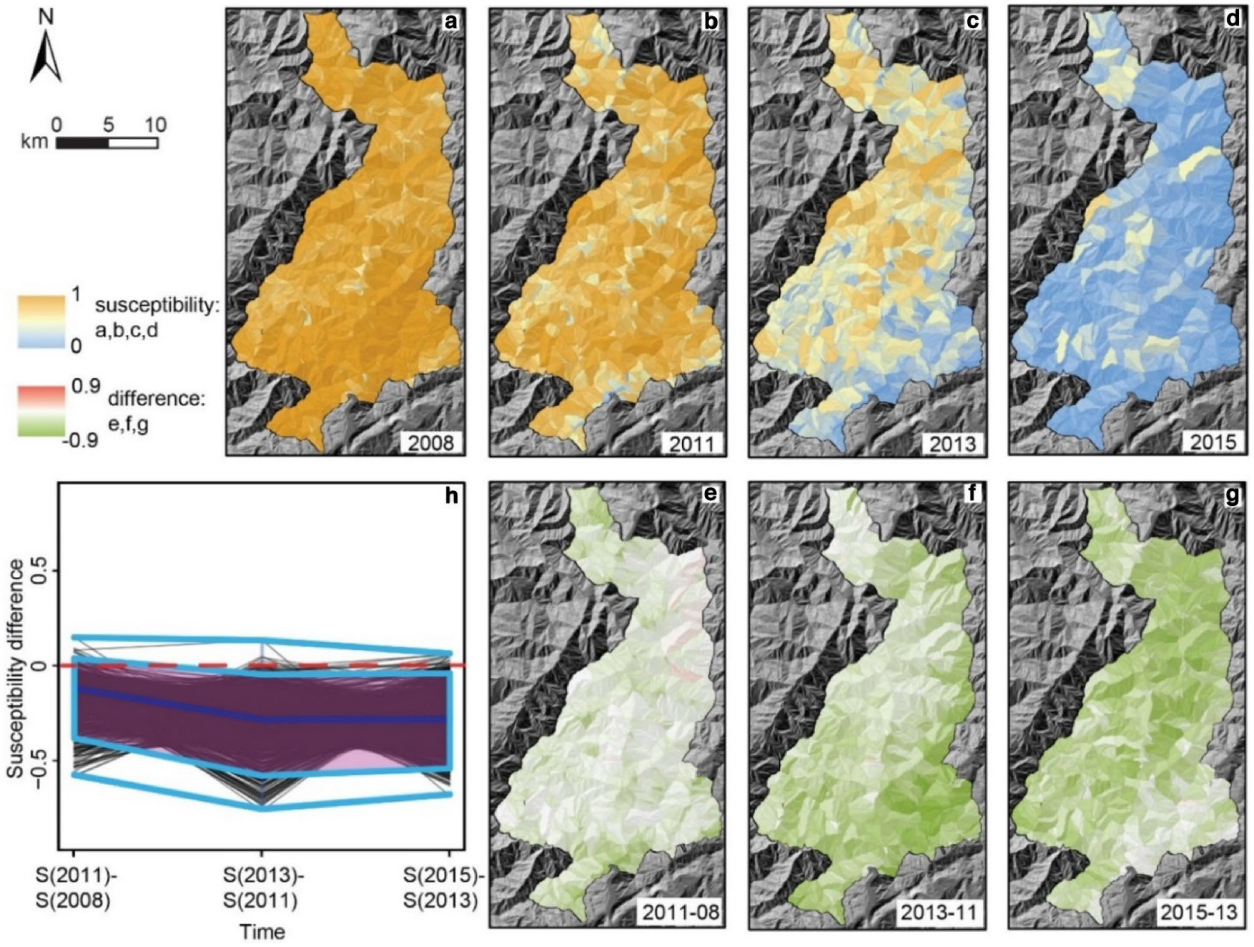
Susceptibility maps across the years (**a–d**) and residual susceptibility maps (**e–g**). The box plot (**h**) tracks the change in susceptibility between scenes for each SU [software: ArcGIS 10.5, www.arcgis.com].

**Figure 5:**
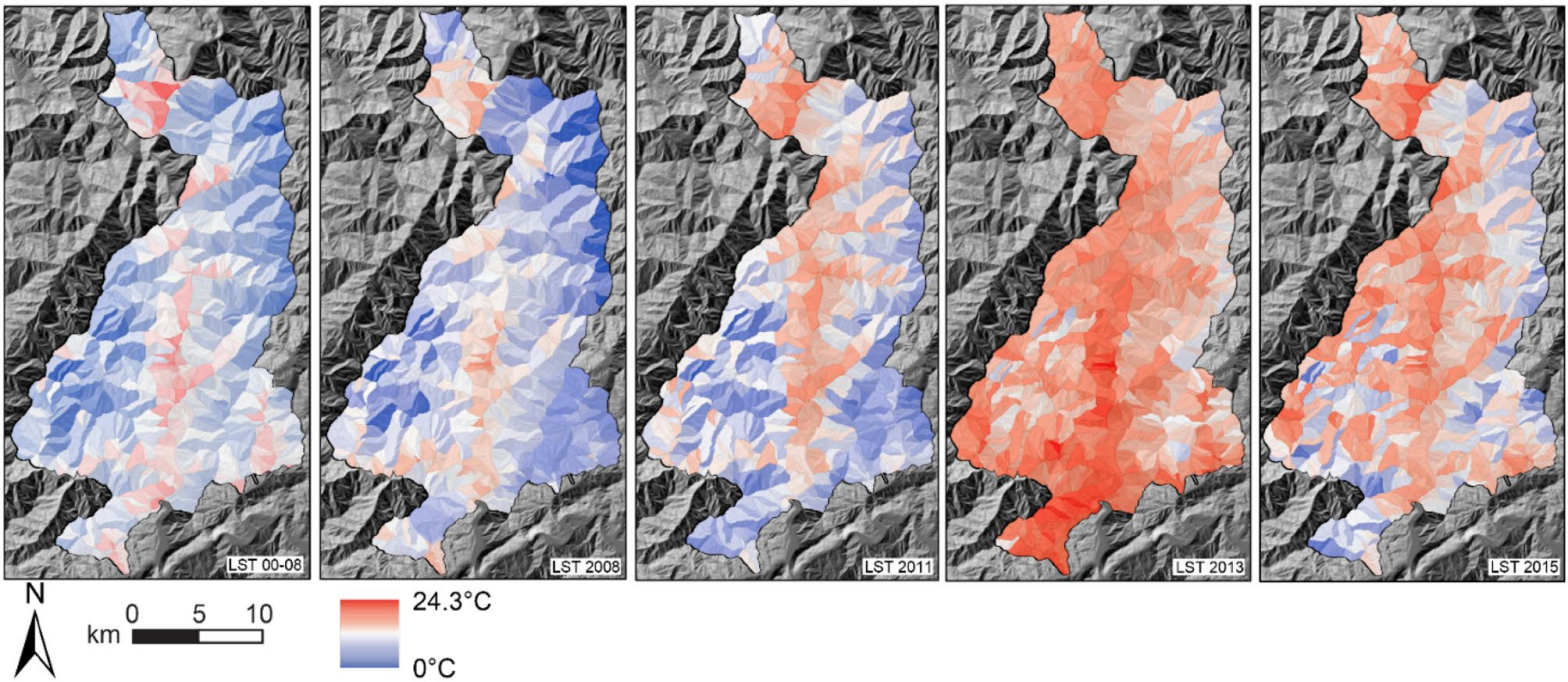
LST data for the WEER-MLI, averaged over the interval between successive image acquisitions. For the 2008 scene, one year of observations prior to the earthquake were considered; for the 2011 scene, data between the imaging date (April 2011) and the earthquake date in (12 May 2008) were taken, and so on. The figure also shows the average LST during eight years before the earthquake (2000–2008), for comparison [software: ArcGIS 10.5, www.arcgis.com].

Additional insight is offered by the spatial patterns of susceptibility and its variation with time (Fig. 4). In 2008 and 2011, high susceptibility is evaluated everywhere in the study area without a distinguishable pattern. Again, this is reasonable given the high PGA values in the whole area and the stochastic character of the early remobilizations. In 2013 and 2015 some pattern emerges, with persistent landslide activity closer to the drainage network. This seems consistent with a mechanism of sediment transfer toward the main stream and with a possible action of stream erosion on fluvial-connected bodies^1,68^. On the other hand, especially if one looks at susceptibility differences between maps (Fig. 4f,g), some similarity emerges with the patterns of LST (Fig. 5 in the Methods). In fact, the slopes with higher susceptibility in 2013 seem to be preferentially located in catchments generally sloping to the southeast (right bank side of the river, that flows generally southward; see also Figure S2); these catchments, at given elevations along the river, appear warmer than those on the opposite bank. The box plot (Fig. 4h) confirms the decay of landslide activity by pointing out that the susceptibility of almost all SUs decreases monotonically with time.

## Discussion and conclusion

By performing a SU-based LSM over multiple scenes of a multitemporal landslide inventory in a rapidly evolving postseismic context, we aimed at explaining the effects of key covariates without a predictive intent. Consistently, we did not aim at excellent performances or at complex models rich in covariates. On the contrary, we wished to highlight the simplest correspondences between patterns/trends and their possible underlying processes.

In particular, we demonstrated that temperature (as LST), a “forgotten” variable in slope stability modeling, does exert a significant control on landslide spatial patterns. What is more, we showed that the role of LST changes with time, from being irrelevant in the coseismic scene to exhibiting a strong and positive correlation once landslide activity has decayed to the pre-earthquake level (2015 scene^5^). We verified that the irrelevance of LST in the coseismic scene was not due to an anomalous LST pattern during that year (Fig. 5 shows that the LST pattern in 2008 is not different from that in the 2000–2008 period), meaning that the thermal signal was indeed overridden by the seismic signal. It could have been interesting to evaluate the LST—landslide susceptibility relationship also in the pre-earthquake period; however, the inventory contains too few landslides in the 2005–2007 interval which, moreover, cannot be associated to a triggering date.

During the post-earthquake transient phase, the effect of temperature is not obvious, but significant effects are shown to emerge progressively. The final, positive and linear correlation is consistent with laboratory observations on the residual shear strength of soils (the sole available in remobilized slides) that do not contain much active clay^26^, and is supported by grain-size analyses^69–71^. It also fits with a process of temperature-enhanced consolidation, typical of poorly consolidated materials^20,21,72^ (as coseismic deposits are, especially the shallow ones). This process would decrease the permeability and increase the likelihood of pore pressure build-up, playing against slope stability. Conversely, a mechanism dominated by evapotranspiration does not seem to hold, as higher temperatures would enhance the water loss to the atmosphere, increasing soil suctions and hence the available mechanical strength. We verified that LST data could provide different insight from that offered by vegetation coverage data (e.g., through the *normalized difference vegetation index*, NDVI). To do so, we ran our model again with NDVI maps^7^ instead of LST maps (see supplementary information). We demonstrated that LST and NDVI data show little collinearity in each year (Figure S3), and verified that in a model with NDVI, the variable effect of the other covariates remains unchanged (Figure S4). The effect of NDVI, however, exhibits less complexity and nonlinearity than that of LST (Figure S5). In fact, while for LST a clear change in significance can be deduced (with a significant positive linear effect emerging progressively, the NDVI effect shows little temporal evolution. This also results in a performance of the model with LST data being somewhat superior compared to that with NDVI data (Figure S6).

In fact, published studies pointed out that the progressive stabilization of coseismic deposits could result from a number of processes, among which, for instance, grain coarsening seems dominant over material exhaustion, consolidation, and revegetation^8^. In particular, grain coarsening by internal erosion of the fine soil fraction during precipitation events was shown to have a beneficial effect on hydraulic conductivity^70,73^, capable alone of increasing landslide-triggering precipitation thresholds and hindering the generation of long-runout events^8^. Removal of the fine soil fraction implies the preferential removal of clay aggregates. These, if in sufficient quantity and with sufficient activity, may dominate not only the soil strength but also its thermal sensitivity owing to their large temperature-dependent volume and strength changes^19^, partly related to their larger water content. The presence of minerals of the smectite group, in particular, would make cooler temperatures conducive to landsliding; their absence (more likely as time goes by) would favor the observed positive correlation between warmer temperatures and slope instability.

Clearly, explaining the thermal effects suggested by geostatistical modeling in terms of physical processes is challenging and requires further analyses in other contexts to seek consistent behaviors. Nevertheless, we believe that our approach, by exploiting SU-based partitioning, GAM, and GEE, is simple yet powerful and has a good potential for systematically investigating the contribution of LST (or other temperature-related variables) to slope instability. LST data, in particular, are freely and widely available thanks to ongoing spaceborne monitoring missions in the infrared band and can be downscaled by identifying relationships with higher-resolution aerial and ground-based observations.

## Methods

### Inventory

The WEER-MLI is the largest and most complete multi-temporal EQIL inventory presently publicly available^58^. It covers an area of 471 km^2^ near the epicenter of the 2008 M_w_ 7.9 Wenchuan earthquake, at the eastern margin of the Tibetan Plateau in Sichuan, China. It comprises 42 catchments with steep slopes and elevations of ~ 450–4000 m a.s.l. The climate is subtropical and monsoonal with a mean annual temperature around 13 °C and precipitation (> 1,250 mm/yr) concentrated in the summer months. The inventory includes ~ 9000 coseismic landslides, covering > 70 km^2^ and representing a considerable proportion of the coseismic mass wasting generated by the earthquake (~ 0.8 km^3^ out of ~ 3 km^3^)^58,74^. Remobilizations of coseismic landslide deposits and postseismic landslides are tracked through time, with maps available from 2005 to 2018^58^. Landslide polygons were delineated on images in the visible spectrum acquired before the monsoon season of each available year (usually in April). Figure 1 shows the abundance of EQILs. Among them, landslides of the slide type prevail, with 8259 polygons mapped in 2008 (coseismic landslides), and 3549, 1041 and 110 polygons, including remobilized coseismic deposits and postseismic landslides in 2011, 2013 and 2015, respectively. We conducted our analysis on this subset of data and excluded pre-earthquake (2005 and 2007) and recent (2017 and 2018) maps owing to the small number of active landslides, insufficient for statistical modeling.

We deem the WEER-MLI appropriate for highlighting the role of LST via LSM. In fact, even though the area features slopes of variable size and shape, the variability in most covariates is not excessive^7^: seismic shaking, lithology, and total precipitation patterns are relatively homogeneous, and so are the slope angles and the elevation of the ridges.

### Mapping units and covariates

Thermal boundary conditions (e.g., solar irradiation, air temperature) are influenced by slope orientation and surface morphology, which are intrinsic characteristics of the SUs. Therefore, working with SUs rather than pixel-based partitions seems the most practical and reasonable approach^75^. We delineated the SUs using *r.slopeunits* software^76^. Specifically, the calculations to produce SUs allowed us to obtain a spatial partition uniquely associated with theoretically susceptible zones to landslides and with objects with geomorphological meaning in slope stability analysis. Then, we intersected the SUs with the inventory to construct a landslide presence/absence dataset, which we used as the target variable in LSM. As the inventory contains landslide polygons, we identified a single point for each polygon that corresponds to the highest location along the landslide scarp. If a SU contained at least one of such points, it was classified as unstable (presence of landslides) as opposed to stable (absence of landslides, no points in the SU).

We used an essential set of covariates together with LST data (Table 1) to perform the susceptibility analysis. We computed the slope inclination using the 25-m DEM available from the Sichuan Bureau of Surveying and Mapping^7^. We accounted for the PGA during the mainshock in 2008 (obtained from the China Earthquake Networks Centre), which reasonably represents the main landslide trigger. Additionally, we accounted for the shape of the SUs through the *maximum distance* (*MD*) from the highest to the lowest point along a SU boundary and computed the *elongation index* (*DA*) as the maximum distance divided by the square root of the SU area. This index represents large SUs when the ratio returns small values, and elongated SU as the ratio increases. Further, we used the LST, which can be defined as “how hot the Earth surface would feel to the touch in a particular location. From a satellite point of view, the surface is whatever it sees when it looks through the atmosphere to the ground” (https://earthobservatory.nasa.gov/global-maps/MOD_LSTD_M).

**Table 1:** List of covariates used in the model.

Name	Abbreviations
Mean peak ground acceleration^7^	PGAµ
Mean slope within the SU^77^	SLPµ
Maximum distance within the SU^54,78^	MD
Max distance/√(SU area)^54,78^	DA
Mean land surface temperature^79,80^	LSTµ

### Land surface temperature

We used TIRS data from Landsat 7 to compute LSTs (Fig. 5). Compared with data from the *Moderate Resolution Imaging Spectroradiometer* (MODIS), which nevertheless highlights consistent patterns, TIRS offers higher spatial resolution (100 m vs. 500–1000 m), which is preferrable for discriminating spatial features. However, Landsat 7 has much longer revisit times (1 month vs. 2 days). We preferred the high spatial resolution over the revisit time as we used averaged data over considerable intervals (i.e., the interval between the date of acquisition of successive images in the inventory: 1–3 years). Moreover, methodological frameworks for deriving LST data from TIRS data series are well codified^79,80^ and can be implemented in GEE^81^. All necessary inputs for data correction also are available in GEE: water vapor content from NCEP/NCAR, emissivity from the ASTER GEDv3, NDVI-based correction for vegetation. Validation of spaceborne against in situ LST data is available for Landsat 5, 7, and 8^79^.

### Modeling strategy

We used a binomial GAM, which is one of the most popular classifiers in landslides susceptibility. A GAM is an extension of a Generalized Linear Model (GLM) with a nonparametric smoothing function^48,49^. GAMs have proven to be highly useful in susceptibility modeling as they allow for the inclusion of non-linear relations between the response (landslides) and quantitative covariates while maintaining a high generalization capacity and interpretability. The GAM formulation in our case can be denoted as:

$$\eta P = \beta_{0} + \, f_{1} \left( {X_{PGA\mu } } \right) + f_{2} \left( {X_{SLP\mu } } \right) + f_{3} \left( {X_{MD} } \right) + f_{4} \left( {X_{DA} } \right) + f_{5} \left( {X_{LST\mu } } \right)$$

where *P* is the probability of landslide occurrence, *η* is the logit link and *f*_*1*_ to *f*_*5*_ represent nonlinear functions for each covariate under consideration.

We recall here that our research aimed at investigating the role of temperature in the failure mechanism of a population of coseismic and postseismic landslides. Therefore, we opted for an explanatory use of the GAM framework, leaving aside considerations on the predictive capacity of the model we built. In other words, we purposely limited the number of covariates to a minimum—featuring only morphological characteristics of the slope as well as the ground motion and the temperature itself—and used all the available data for fitting, without bootstrapping random subsets for cross-validation routines. We measured the goodness of fit via common performance metrics for binary classifiers. Specifically, we used ROC and PR curves and their AUC to test whether the explanatory power^59^ of the fitted binomial GAM was suitable to make an interpretation of each covariate role.

## Supplementary Information

Supplementary Information.

## Data Availability

This work was built upon freely available datasets and tools.
