# Supplementary material for: Surface temperature controls the pattern of post-earthquake landslide activity
Source: Sci Rep. 2022 Jan 19;12:988. doi: 10.1038/s41598-022-04992-8 (PMC8770705; doi:10.1038/s41598-022-04992-8)
Supplement: Supplementary file 1 — Supplementary Information. [file 41598_2022_4992_MOESM1_ESM.pdf]

Supplementary information

Surface temperature controls the pattern of post-earthquake landslide activity

Marco Loche[1], Gianvito Scaringi[1,*], Ali P. Yunus[2], Filippo Catani[3], Hakan Tanyaş[4], William Frodella[5], Xuanmei Fan[2], Luigi Lombardo[4]

[1] Institute of Hydrogeology, Engineering Geology and Applied Geophysics, Faculty of Science, Charles University, Prague, Czech Republic

[2] State Key Laboratory of Geohazard Prevention and Geoenvironment Protection, Chengdu University of Technology, Chengdu, China

[3] Department of Geosciences, University of Padova, Padova, Italy

[4] Faculty of Geo-Information Science and Earth Observation (ITC), University of Twente, Enschede, Netherlands

[5] Department of Earth Sciences, University of Florence, Florence, Italy

* Corresponding author: gianvito.scaringi@natur.cuni.cz

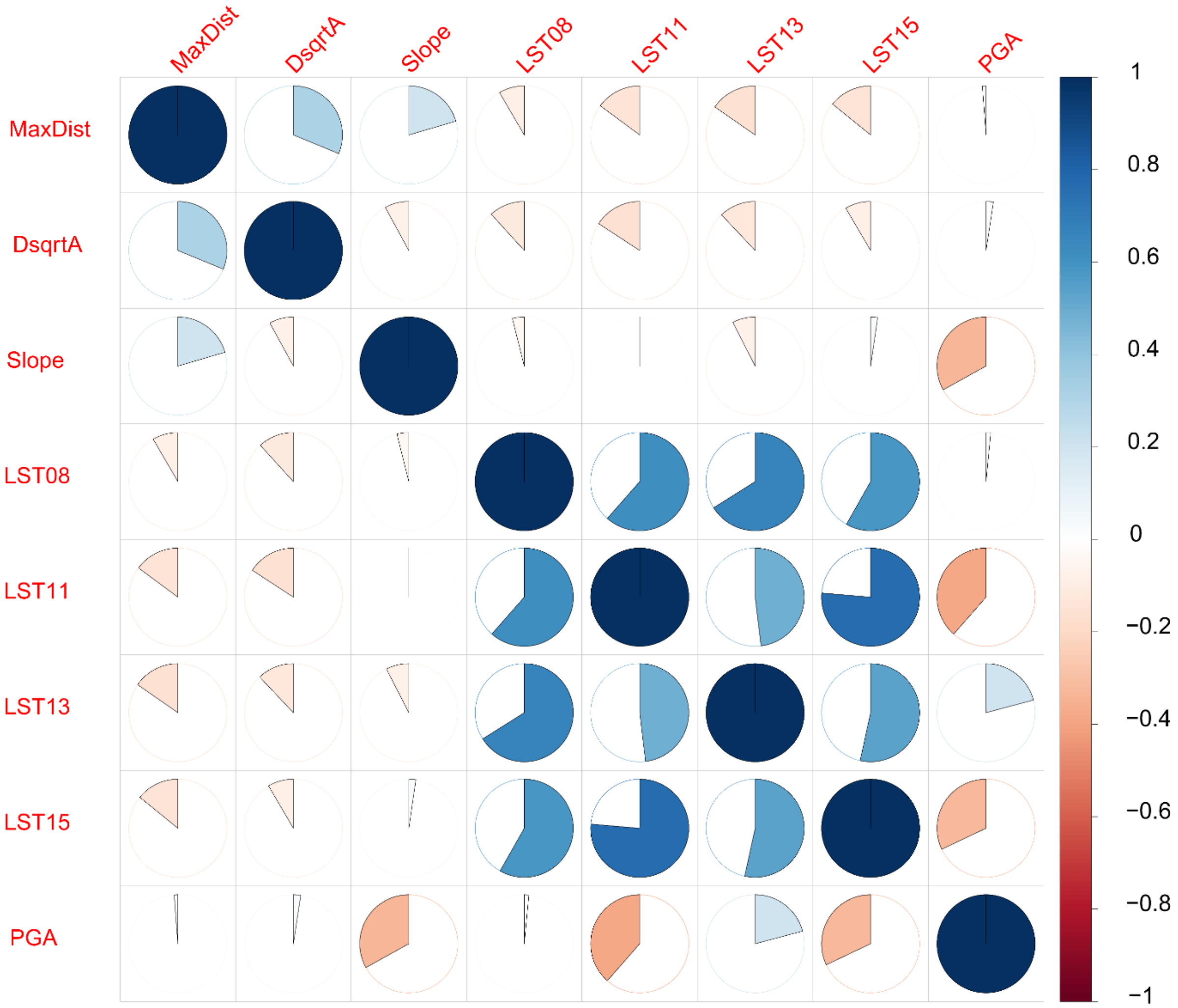

Figure S1. Collinearity between the variables used in the model. Note that LST08, LST11, LST13, and LST15 were used to obtain the susceptibility maps of 2008, 2011, 2013, and 2015, respectively [software: R 3.6.3, https://cloud.r-project.org/].

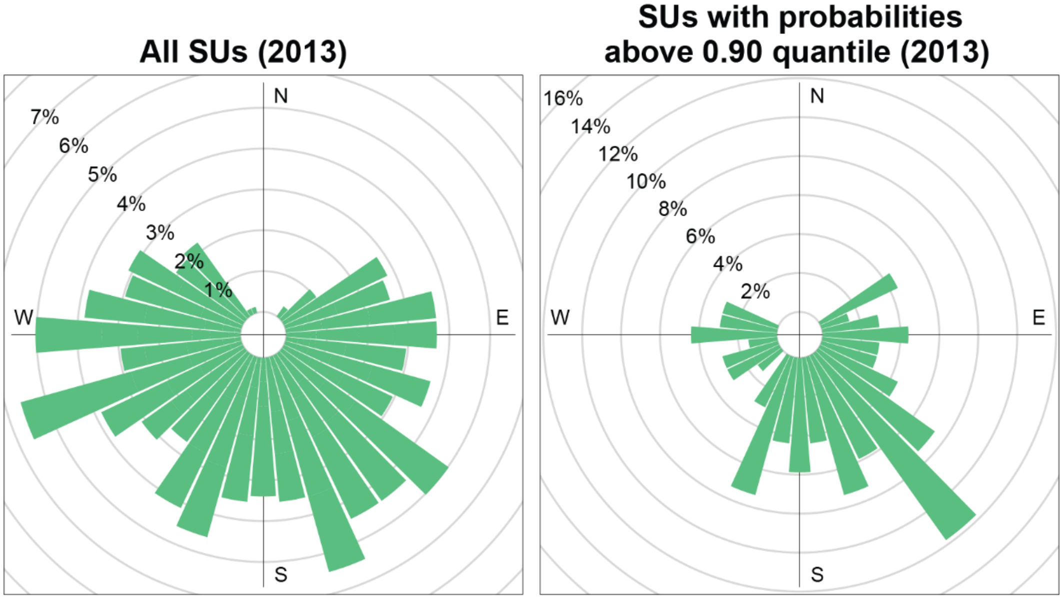

Figure S2. Orientation of all slope units in the study area (left) and slope units with the 10% highest landslide susceptibility in 2013 (right) [software: R 3.6.3, https://cloud.r-project.org/].

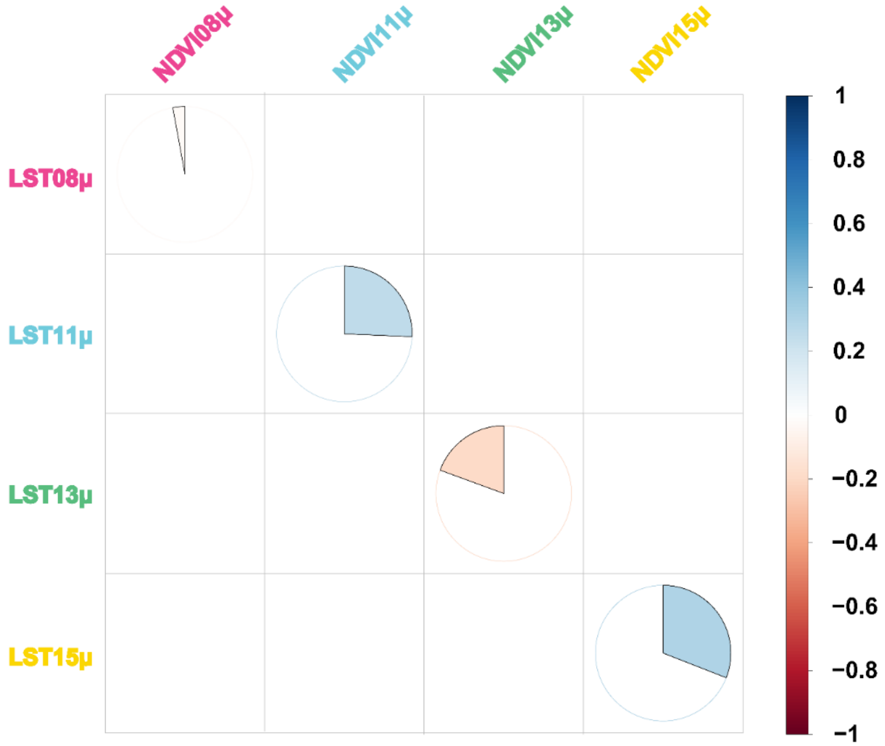

	NDVI08µ	NDVI11µ	NDVI13µ	NDVI15µ
LST08µ	-0.03			
LST11µ		0.26		
LST13µ			-0.19	
LST15µ				0.31

Figure S3. Collinearity between Land Surface Temperature (LST) and Normalized Difference Vegetation Index (NDVI) data for each year. NDVI data are the same as those used by Fan et al. (2021, https://doi.org/10.1029/2020GL090509) [software: R 3.6.3, https://cloud.r-project.org/].

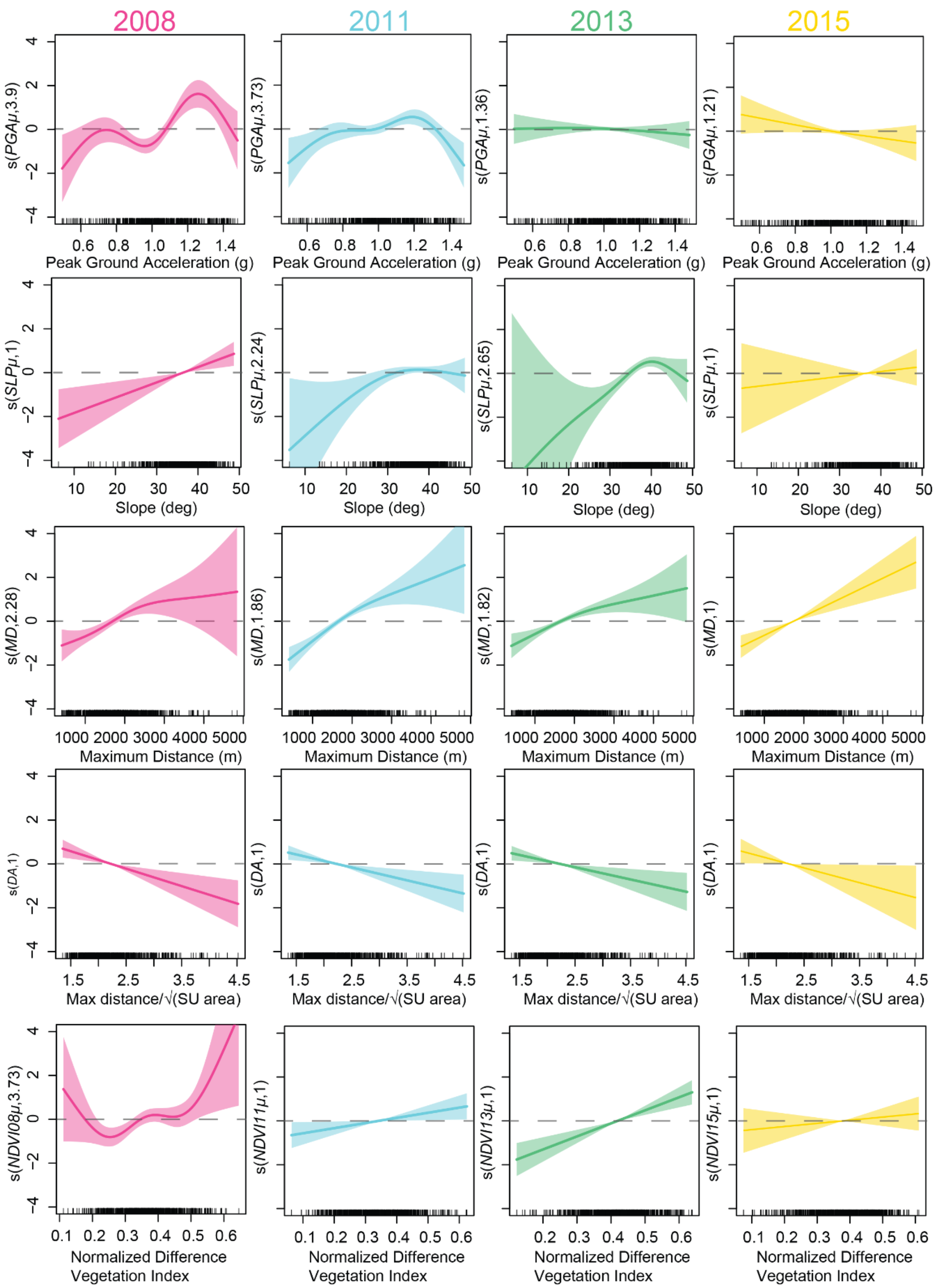

Figure S4. Variable effect of the covariates over time for a landslide susceptibility model analogous to that described in the article (see Figure 3) but with NDVI instead of LST data [software: R 3.6.3, https://cloud.r-project.org/].

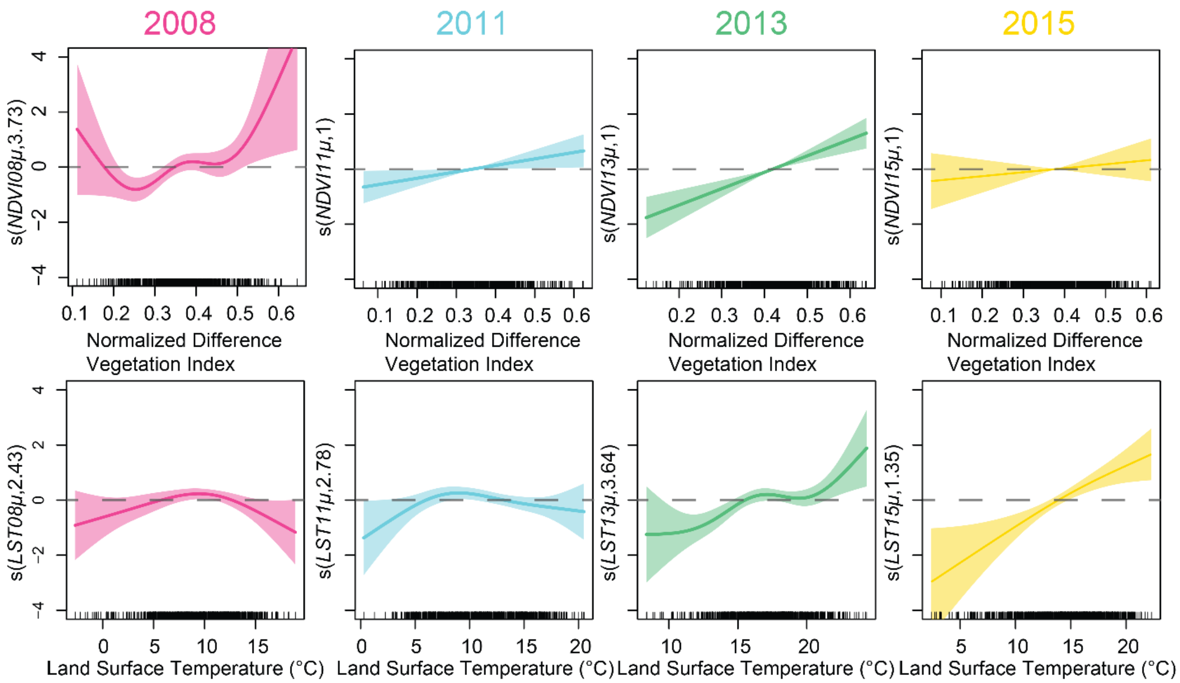

Figure S5. Comparison between variable effects of NDVI (top) and LST (bottom) [software: R 3.6.3, https://cloud.r-project.org/].

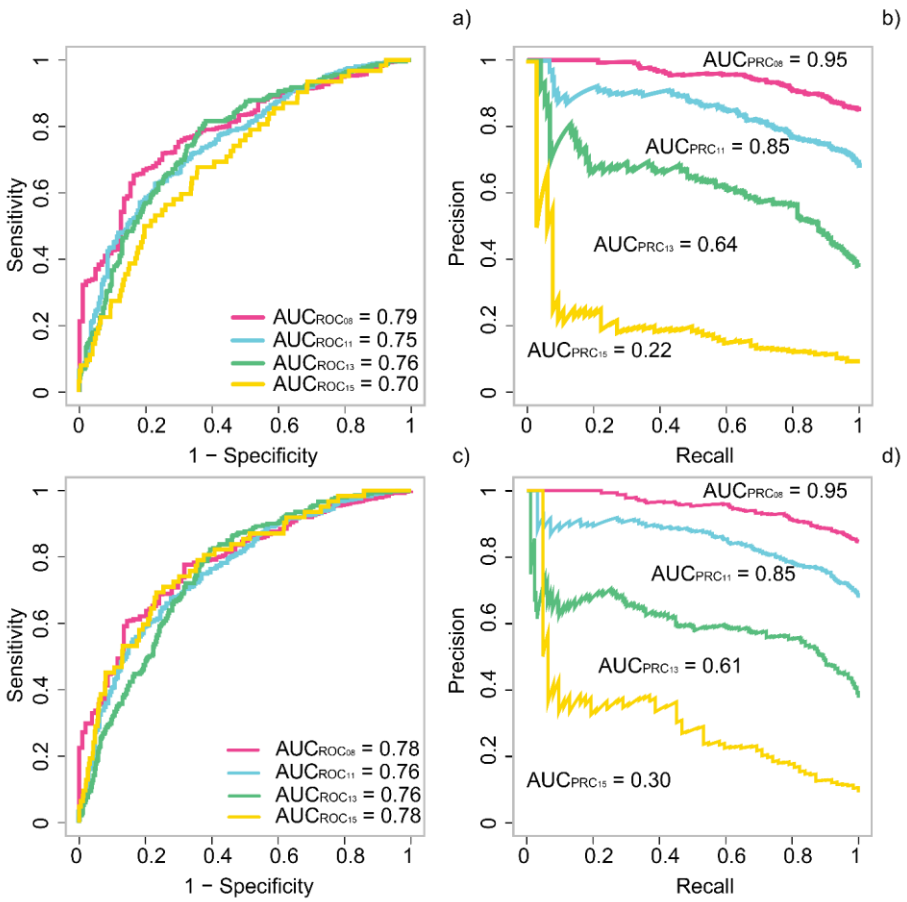

Figure S6. Performance of the model constructed by replacing LST with NDVI data (a, b) and of the original model described in the article (c, d) (see also Figure 2). (a, c) show the ROC curves while (b, d) the PR curves [software: R 3.6.3, https://cloud.r-project.org/].
